# Designing of a Novel Multi-Antigenic Epitope-Based Vaccine against *E. hormaechei*: An Intergraded Reverse Vaccinology and Immunoinformatics Approach

**DOI:** 10.3390/vaccines10050665

**Published:** 2022-04-22

**Authors:** Thamer H. Albekairi, Abdulrahman Alshammari, Metab Alharbi, Amal F. Alshammary, Muhammad Tahir ul Qamar, Asad Ullah, Muhammad Irfan, Sajjad Ahmad

**Affiliations:** 1 Department of Pharmacology and Toxicology, College of Pharmacy, King Saud University, P.O. Box 2455, Riyadh 11451, Saudi Arabia; thalbekairi@ksu.edu.sa (T.H.A.); abdalshammari@ksu.edu.sa (A.A.); mesalharbi@ksu.edu.sa (M.A.); 2 Department of Clinical Laboratory Sciences, College of Applied Medical Sciences, King Saud University, Riyadh 11451, Saudi Arabia; aalshammary@ksu.edu.sa; 3 Department of Bioinformatics and Biotechnology, Government College University Faisalabad, Faisalabad 38000, Pakistan; 4 Department of Health and Biological Sciences, Abasyn University, Peshawar 25000, Pakistan; asadullahaup@gmail.com (A.U.); sajjad.ahmad@abasyn.edu.pk (S.A.); 5 Department of Oral Biology, College of Dentistry, University of Florid, Gainesville, FL 32611, USA; irfanmuhammad@ufl.edu

**Keywords:** antibiotics resistant, *E. hormaechei*, reverse vaccinology, docking, molecular dynamic simulation

## Abstract

*Enterobacter hormaechei* is involved in multiple hospital-associated infections and is resistant to beta-lactam and tetracycline antibiotics. Due to emerging antibiotics resistance in *E. hormaechei* and lack of licensed vaccine availability, efforts are required to overcome the antibiotics crisis. In the current research study, a multi-epitope-based vaccine against *E. hormaechei* was designed using reverse vaccinology and immunoinformatic approaches. A total number of 50 strains were analyzed from which the core proteome was extracted. One extracellular (curlin minor subunit CsgB) and two periplasmic membrane proteins (flagellar basal-body rod protein (FlgF) and flagellar basal body P-ring protein (FlgI) were prioritized for B and T-cell epitope prediction. Only three filtered TPGKMDYTS, GADMTPGKM and RLSAESQAT epitopes were used when designing the vaccine construct. The epitopes were linked via GPGPG linkers and EAAAK linker-linked cholera toxin B-subunit adjuvant was used to enhance the immune stimulation efficacy of the vaccine. Docking studies of the vaccine construct with immune cell receptors revealed better interactions, vital for generating proper immune reactions. Docked complexes of vaccine with MHC-I, MHC-II and Tool-like receptor 4 (TLR-4) reported the lowest binding energy of −594.1 kcal/mol, −706.7 kcal/mol, −787.2 kcal/mol, respectively, and were further subjected to molecular dynamic simulations. Net binding free energy calculations also confirmed that the designed vaccine has a strong binding affinity for immune receptors and thus could be a good vaccine candidate for future experimental investigations.

## 1. Introduction

Resistance to antimicrobials by bacteria is an alarming public health issue at present [1]. According to the World Health Organization (WHO), antimicrobial resistance (AMR) is defined as “a global health security threat that requires action across society and government sectors as a whole”. As per a report by the Center for Disease Control and Prevention (CDC), there is a direct link between healthcare cost and AMR, which is as high as $20 billion per year [2]. Furthermore, the cost incurred for lost productivity was $35 billion a year in just the United States of America alone [3]. Bacteria that are frequently isolated from clinical samples include *Klebsiella* species, *Escherichia coli*, and *Enterobacter* species [4]. In *Enterobacteriaceae*, AMR reached a dangerous level and several community-associated and healthcare-related infections are hard to treat [5].

In several research studies, the rise of carbapenemase-producing *Enterobacteriaceae* (CPE) was reported in recent years. Infections caused by CPE in the US are usually healthcare-associated, although community-associated infections are now becoming evident [6]. The threat of CPE is significant as Carbapenems is used for the treatment of infections which are caused by extended-spectrum β-lactamase-producing Enterobacteriaceae (ESBL-E) [7].

*Enterobacter hormaechei* is a gram-negative bacterial pathogen responsible for nosocomial infections [8]. In recent years, the pathogenic *E. hormaechei* was isolated from piglets and foxes [9]. The bacterial strains showed different degrees of resistivity to amikacin, azlocillin, maxolactam, cefotaxime, ceftazidime, ceftriaxone, chloramphenicol, gentamicin, mezlocillin, tobramycin, piperacillin, trimethoprim-sulfamethoxazole, sulfisoxazole, thienamycin, and trimethoprim [10,11,12]. All these strains were also resistant to nitrofurantoin and most of them were resistant to cefoxitin, ampicillin, and cephalothin. [13]. Therefore, a long lasting and effective therapy is needed to counter the *E. hormaechei* infections.

The process which involves the discovery of antigens from a genome is called reverse vaccinology [14]. From its first application of a successful vaccine developed against Neisseria *meningitidis* group B, this approach progressively evolved and was accepted as an attractive method of vaccine discovery [15]. This discovery led to the development of vaccines against different bacterial and other pathogens [16,17]. Existing reverse vaccinology approaches comprise the comparative in silico analyses of multiple genome sequences to identify the conserved antigens within a heterogeneous pathogen population; they are also used for the identification of antigens that are distinctive to pathogenic isolates but not present in commensal strains [18]. Moreover, transcriptomics and proteomic datasets are integrated into a selection process that yields lists of antigens to be tested in animal models, thus decreasing the time and costs of downstream analyses [19]. This study specifically aimed to explore the core proteome of *E. hormaechei* complete strains for designing a novel multi-epitope-based vaccine against *E. hormaechei* using subtractive proteomics, reverse vaccinology, and immunoinformatics to identify suitable candidates for vaccine design. The identified vaccine targets and multi-epitopes vaccine design are experimentally tested against the bacterial pathogen, using in vivo and in vitro studies. Our results will serve as a pioneer work that attempts to identify an immunogenic vaccine model against *E. hormaechei* infections.

## 2. Research Methodology

**Figure 1:**
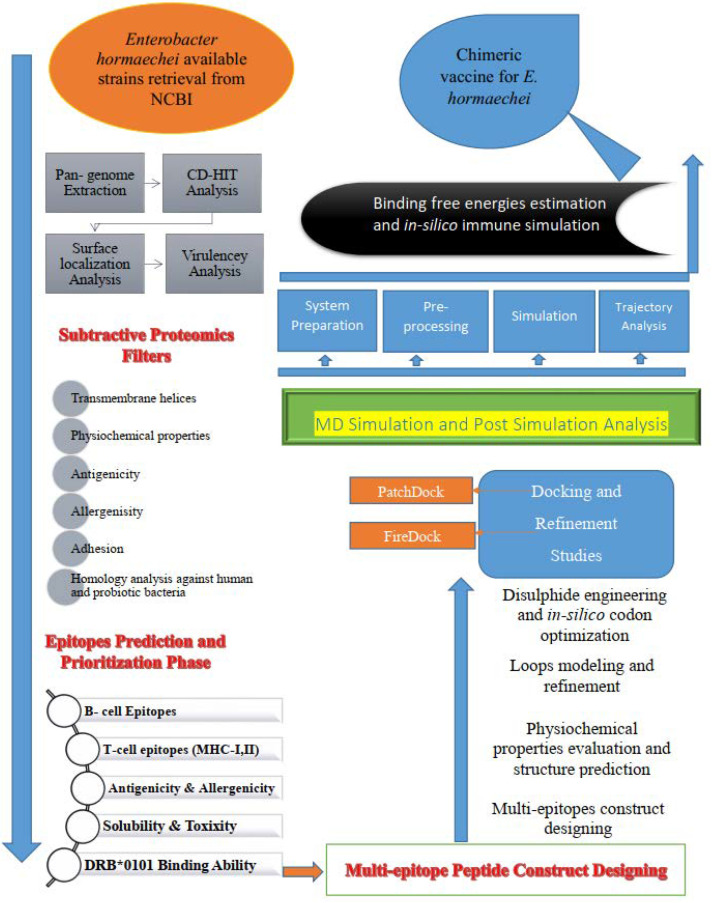
In silico approach used for the design of multi-epitope vaccine against *E. hormaechei*.

The complete flow chart used for the design of an in silico multi-epitope-based vaccine targeting *E. hormaechei* is presented in Figure 1. 

### 2.1. Retrieval of Complete Proteome and Core Proteome Identification of E. hormaechei

In the first phase of the study, a total number of 50 fully sequenced genomes were extracted from the National Center for Biotechnological Information (NCBI) database [20] and subjected to core proteome identification using the bacterial pan-genome analysis (BPGA) pipeline [21]. BPGA is an ultra-fast software and can provide all-inclusive pan-genome analysis of bacterial species i.e., profile of core genome and core/pan plot phylogeny [21]. After retrieval of the core proteome from the complete proteome of *E. hormaechei*, the core proteome was subjected to CD–HIT analysis using the official online CD–HIT webserver [22]. CD–HIT is an online webserver developed by Dr. Weizhong Li, mainly used for the comparing and clustering of proteins and nucleotide sequences [23]. In the CD–HIT analysis, non-redundant protein sequences were extracted [22]. The non-redundant protein sequences were further considered for surface localization analysis and surface-localized proteins were identified using an online PSORTb server [24]. In subcellular localization analysis, only outer membrane, periplasmic membrane, and extracellular membrane protein sequences were considered and cytoplasmic and membrane protein sequences were discarded as cytoplasmic proteins can only be used as drugs targets [25]. Surface proteins are exposed to the host immune system and can generate proper immune reactions and can, therefore, be used as suitable vaccine candidates [26]. Virulent proteins have the ability to cause infection and can be used as good vaccine candidates to produce a proper immune response [27]. To identify virulent proteins, the virulent-factor database (VFDB) [28] approach was used and only those hit having a bit score greater than 100%, as well as a sequence identity greater than 30% were selected, while the remaining all non-virulent proteins were discarded [29]. After VFDB analysis, all the virulent proteins were checked for helices using TMHMM-2.0 webserver [30]; the selection criteria were set as: (i) proteins having more than one transmembrane should be discarded, and (ii) only those proteins having zero and one transmembrane helices should be selected [31]. Furthermore, physiochemical properties analysis and homology checks against human (taxid: 9606) and three normal microbiota species *Lactobacillus. casei* (taxid: 1582), *L. rhamnosus* (taxid: 47715) and *L. johnsonii* (taxid: 33959) were performed. This task was achieved via the online BLASTp webserver [32]. After all the above checks, proteins were shortlisted and considered as good vaccine candidates [33].

### 2.2. B- and T-Cell Epitope Mapping

Several bacterial species that cause infections in humans are counteracted by humoral and cellular immunity [34]. Herein, B-cell epitopes were predicted from shortlisted proteins using immune epitope database analysis and a resource (IEDB) webserver [35]. In B-cell epitope selection, linear B-cell epitopes were predicted choosing Bepipred Linear Epitope Prediction 2.0 on the IEDB server [36]. Subsequent to B-cell epitope prediction, T-cell epitopes were then predicted in order to generate cellular immunity [37]. In T-cell epitopes, both MHC-I and MHC-II epitopes were predicted using the epitope analysis resources tab in the IEDB webserver; all the epitopes were considered good based on the lowest percentile rank [38].

### 2.3. Construction and Processing of the Multi-Epitope Vaccine Model

A multi-epitope-based vaccine construct consists of several probable antigenic immunodominant epitopes; hence, such a vaccine can be considered an ideal approach to tackling many bacterial diseases [39]. During the multi-epitope vaccine construction phase, all the probable antigens that are non-allergic, non-toxin and have good water-soluble capacity were linked with each other through specific GPGPG linkers [40], and fused with an adjuvant (Cholera toxin B-subunit adjuvant) via an EAAAK linker in order to make the vaccine construct more potent [41]. The designed multi-epitope vaccine construct was further evaluated for physiochemical property analysis using the ProtParam Expassy tool [42]. After physiochemical analysis, the 3D dimensional structure was predicted through the Scratch Protein Predictor, which is an online tool [43]. Using SOLpro and ProSA-WEB, solubility and the Z-score of the designed vaccine were predicted [44,45]. Furthermore, loop re-modeling and refinement of the vaccine construct 3D structure was performed using the GalaxyWEB tool [46]. In order to avoid structure instability, the disulfide engineering approach was applied using the Design 2.0 webserver [47]. Additionally, in silico cloning and codon optimization process were performed using SnapGene software [47]. Firstly, the sequence of the multi-epitope-based vaccine model was reverse transcribed to DNA using the JCat tool [48] and then reverse transcribed DNA was cloned into *E.coli* pET-28a (+) vectors to ensure the best expression system [49]. World and different country-wise population coverage analysis was also performed using the IEDB server [50].

### 2.4. Molecular Interaction Analysis Study

Molecular docking studies have been the most important approach in studying vaccine interactions with immune receptors [51]. This analysis mainly allows the prediction of interactions between a vaccine and immune cell receptors. Herein, the online Cluspro 2.0 webserver was utilized for docking studies [52]. Firstly, the immune receptors MHC-I(1L1Y), MHC-II(1KG0), and TLR-4 (4G8A) were retrieved from the protein data bank using their specific PDB:IDs [53]. In each docking, the number of interactions was set at 20. 

### 2.5. Molecular Dynamic (MD) Simulation and Binding Free Energy Calculation

The molecular dynamic simulation approach is a computer-based in silico approach for determining the dynamic behavior of docked molecules [54]. In the current study, to understand the stability, structural quality and dynamic behavior of the docked molecules, MD simulation was run over a time period of 250 nanoseconds (ns) [55]. The MD analysis was completed in three stages, which consisted of parameterization of docked molecules, the pre-processing phase, and the production of the simulation phase. In the initial phase, an antechamber module of AMBER 20 software was used to set several parameters for the designed vaccine and immune cell receptors [56]. All the docked molecules were solvated in a 12 Å TIP3P solvation box and achieved via the AMBER Leap module. The Ff14SB force field was utilized to describe both the designed vaccine and the immune cell receptor molecules [57].

The net binding free energies for the vaccine and immune receptors were computed through MMPBSA.py module in AMBER20 [58]. The average values of these net binding free energies were assessed as the overall binding free energy of the systems. Mathematical-based analysis of MMPB/GBSA was performed by following published protocol [59]. Computing of net binding free energies for all three components was completed by either Poisson Boltzman (MM-PBSA) or Generalized Born (MM-GBSA) [60].

### 2.6. Immune Simulation

The designed vaccine construct was also subjected to the online C-ImmSim webserver [61] in silico to check the ability of the vaccine to induce different immune responses inside the host cells [62]. The C-ImmSim server functions using a position-specific scoring matrix (PSSM) and a machine learning base to analyze the response of the immune system against the administered designed vaccine [63]. 

## 3. Results and Discussion 

### 3.1. Complete Proteome Retrieval and Identification of Core, Non-Redundant, Surface-Localized, Virulent Proteins

**Figure 2:**
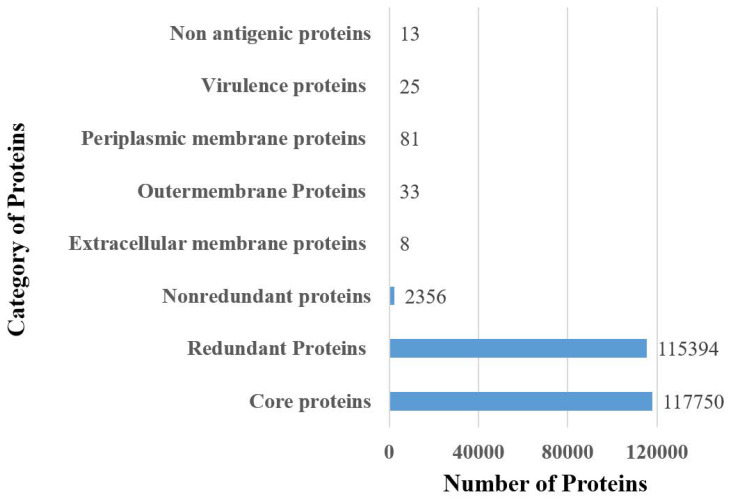
Number of proteins obtained at each step of subtractive proteomics.

**Figure 3:**
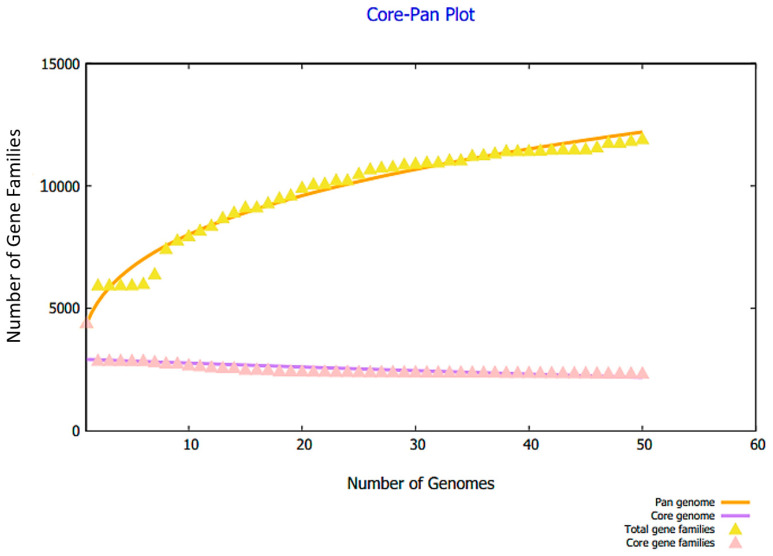
Pan-core plot of 50 strains of *E. hormaechei*.

In the complete proteome retrieval phase, the proteomic data of 50 fully sequenced strains of *E. hormaechei* were retrieved. The core proteome consists of 117,750 core proteins and 115,394 redundant proteins; 2356 proteins were non-redundant, as mentioned in Figure 2. Among the 2356 non-redundant proteins, eight were extracellular, thirty-three were outer membrane, and eighty-one were found as a periplasmic membrane, as shown in Figure 2. In total, 122 different surface-localized proteins were reported. Twenty-five were found to be virulent proteins, while among the twenty-five virulent proteins, thirteen were found to be non-antigenic, as also seen in Figure 2. The genome size of each stain is shown in Figure 3. 

### 3.2. Physiochemical Properties, Transmembrane Helices, Allergenicity and Homology Analysis

**Figure 4:**
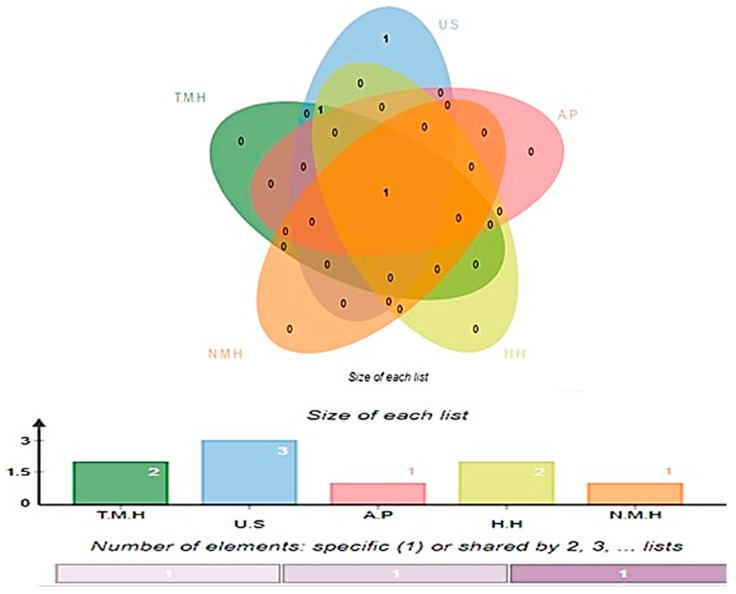
Discarded proteins having transmembrane helices (T.M.H), unstable (U.S), allergic proteins (A.P) human homologous (H.H) and normal microbiota homologous (N.M.H).

In the physiochemical property evaluation, three protein sequences were found unstable, while in the transmembrane helices filter, two proteins were revealed to have more than one transmembrane helix, and one protein was found to be allergic in nature. In the homology analysis, two proteins were significantly similar to human and one showed similarity to three normal microbiota species, *L. casei* (taxid: 1582), *L. rhamnosus* (taxid: 47715) and *L. johnsonii* (taxid: 33959) [64]; all results are shown in Figure 4. 

### 3.3. Prioritization of Potential B- and T-Cell (MHC-I, MHC-II) Epitopes

Humoral immunity is mainly mediated by activated plasma and B-cells; in the epitope prediction phase, B-cell epitopes were, therefore, predicted. One extracellular, curlin minor subunit (CsgB); two periplasmic flagellar basal-body rod proteins (FlgF); and a flagellar basal-body P-ring protein (FlgI) were shortlisted for B-cell epitope prediction. All these help the bacteria in mobility, adhesion, and virulence. One epitope from the curlin minor subunit CsgB protein, two epitopes from the flagellar basal-body rod protein, and three epitopes from the flagellar basal-body P-ring protein were predicted. All predicted epitopes are listed in Table 1. Furthermore, the predicted B-cell epitopes were subjected to T-cell epitope prediction. In T-cell epitope prediction, both MIH-C class-I and II were considered and only those epitopes having the least percentile score were shortlisted, as tabulated in Table S1. 

### 3.4. Multi-Epitope-Based Vaccine Design and Processing

**Figure 5:**
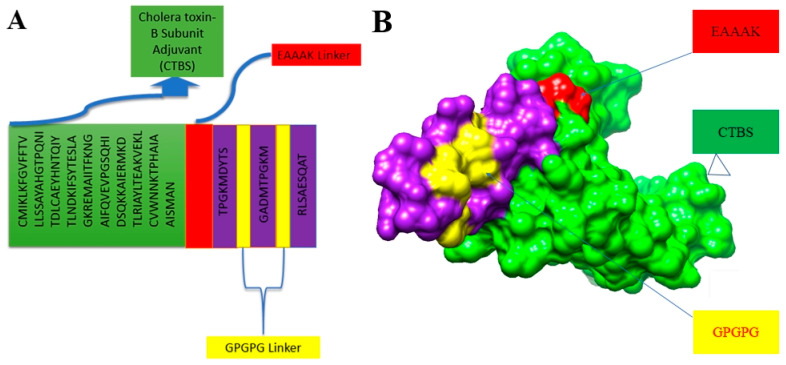
(**A**) Schematic representation of multi-epitope-based vaccine (**B**) three-dimensional structure of designed vaccine. Yellow color represents GPGPG linkers. Light green color is of cholera toxin B subunit adjuvant, while red color represents EAAAK linker.

Designing multi-epitope-based vaccines is a promising approach as it reduces the limitations associated with pasture vaccinology methods as well as sub-unit vaccines. Furthermore, due to the limited antigenic ability of single peptide-based vaccines, multi-epitope-based vaccines proved to stimulate proper immune responses [65]. The multi-epitope-based vaccine was constructed by joining predicted epitopes from the shortlisted proteins. The epitopes were joined together through GPGPG linkers. The designed vaccine construct was further linked with a cholera toxin B-subunit adjuvant to enhance the potency and immunogenicity of the designed vaccine. The adjuvant was linked to the N-Terminal site using an EAAAk linker. The GPGPG and EAAAK linkers reduced Beta turn and increase the alpha region, hence making the vaccine construct less flexible [66]. The schematic diagram of the designed vaccine construct, along with the GPGPG and EAAAK linkers, is represented in Figure 5A, while the 3D structure of the designed vaccine construct is shown in Figure 5B. Additionally, the physiochemical properties of the designed vaccine construct were successfully predicted, as the construct consists of 166 amino acid residues having molecular weight (17.97 kDa), a theoretical pI (8.82), an instability index of 31.84, and an aliphatic index of 73.61. 

### 3.5. Loop Modeling, Refinement, Disulfide Engineering and In Silico Codon Optimization

As too many loops in the protein structure may affect the stability and make it more flexible, an in-loop modeling process consisting of several runs of loops was performed. Three loops of amino acid residues were considered for loop modeling; MET1-GLY7, GLY54-VAL 73, and SER-GLY157. After loop re-modeling, the first model from the predicted models was selected based on the best structure values. A list of all generated values for each predicted model are shown in Table 2.

**Figure 6:**
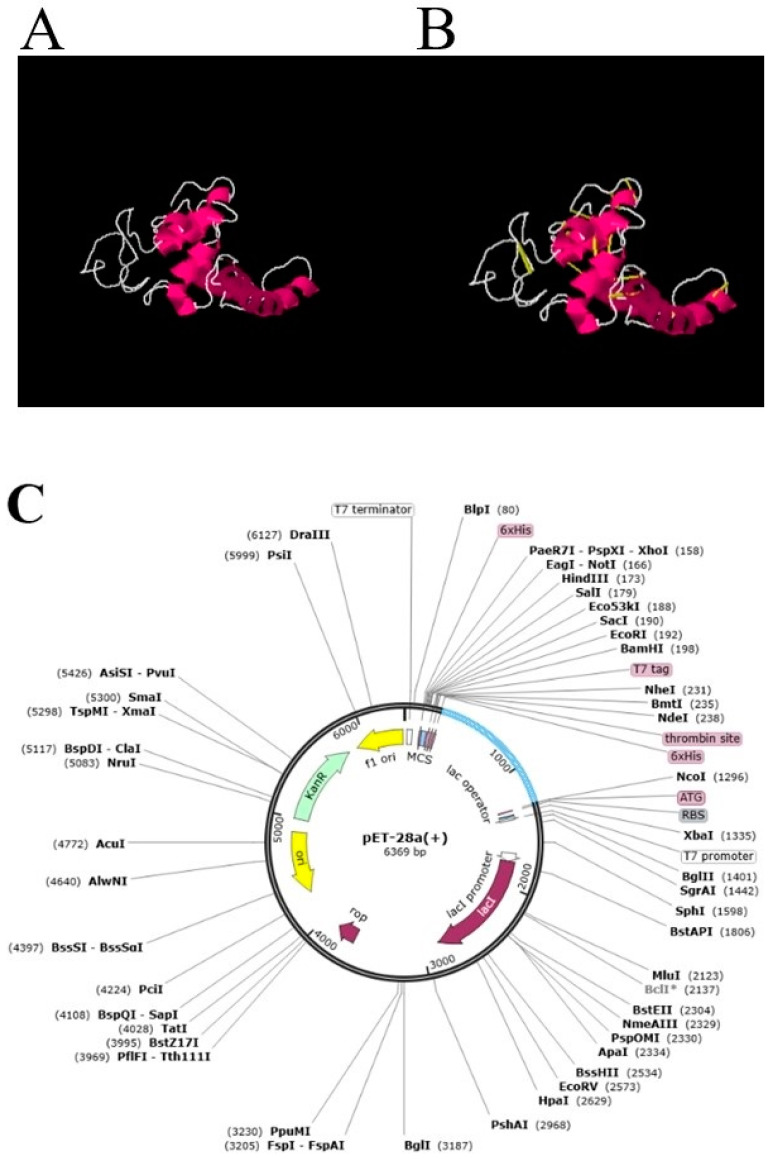
(**A**). Wild structure of designed vaccine, (**B**). Mutated structure of the vaccine. Yellow color stick shows mutation by cysteine amino acid. (**C**). in silico cloned pET28a vector.

Subsequently, disulfide engineering of the designed vaccine construct was accomplished for nineteen pairs of amino acid residues, as indicated in the Table 3. Figure 6A,B represent original and mutant structures of the designed vaccine construct. 

Additionally, computer-based cloning of the multi-epitope vaccine construct was achieved in a plasmid pET28a (+). Reversed transcribed and optimized codon of the designed vaccine construct was performed as per *E. coli* K12 strains as indicated in Figure 6C. The codon adaptation index (CAI value) of the designed vaccine was noted as 0.9574 which indicates best expression, while the GC content was 50.50%, which is also equal to the *E. coli* K12.

**Figure 7:**
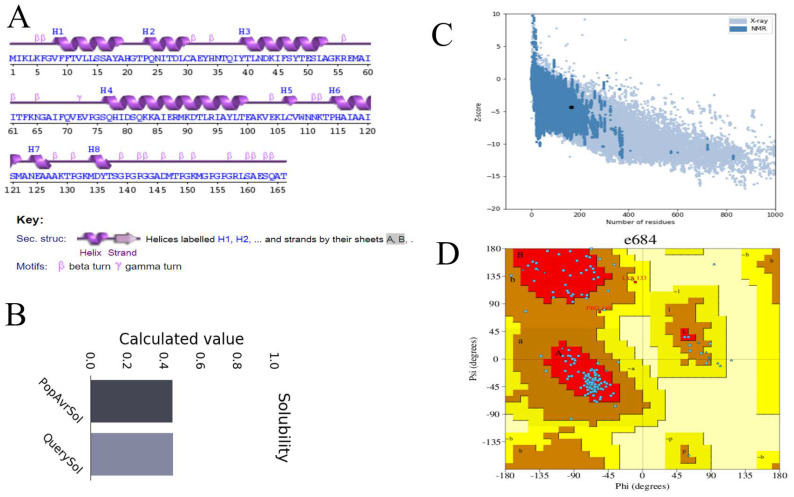
(**A**) Secondary structure, (**B**) computed solubility, (**C**) Z-score graph and (**D**) Ramachandran plot.

The secondary structure of the designed vaccine construct has seventy-three (44.0%) alpha helix residues, shown in Figure 7A. The multi-epitope 3D vaccine structure has 87.9% of residues in the Ramachandran plot favored, 8.3%, in additionally allowed regions, 11.3% in generously allowed regions, and 0.7% residues in disallowed regions, as indicated in Figure 7D. The z-score of the vaccine is −4.3, as indicated in Figure 7C and the solubility score is 0.4, as indicated in Figure 7B. 

### 3.6. World and Country Wise Population Coverage Analysis of Vaccine 

**Figure 8:**
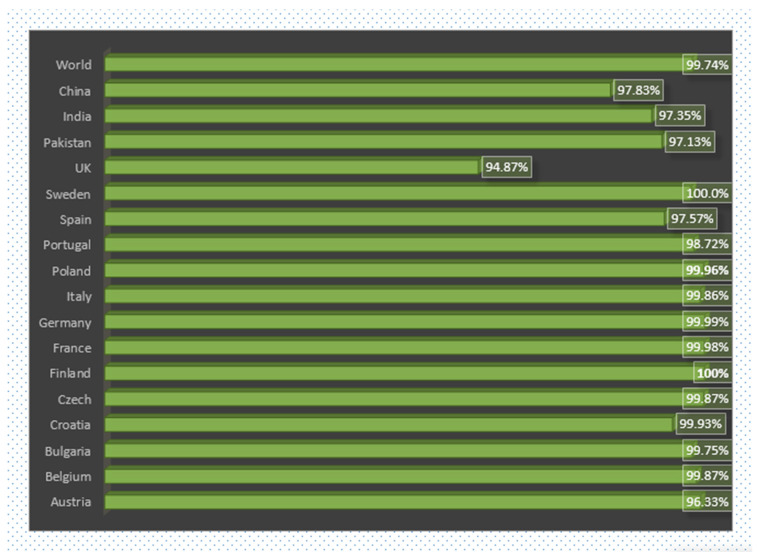
World and different country-wise combined MHC-I and MHC-II coverage of designed vaccine.

The shortlisted epitopes for designing a multi-epitope vaccine construct shows promising potency and good worldwide and several country-wise population coverage. A combined MHC-I and MHC-II population coverage method was used for the final selected epitopes against geographic areas around the world. The findings revealed that MHC-I and MHC-II have the largest worldwide population coverage, at 99.74%. Furthermore, according to the webserver, the anticipated values for China, India and other countries are shown in Figure 8. We conclude that certain epitopes may be viewed as possible aspirants and should be explored for inclusion in the creation of a multi-epitope vaccine design.

### 3.7. Docking and Simulation Analysis

**Figure 9:**
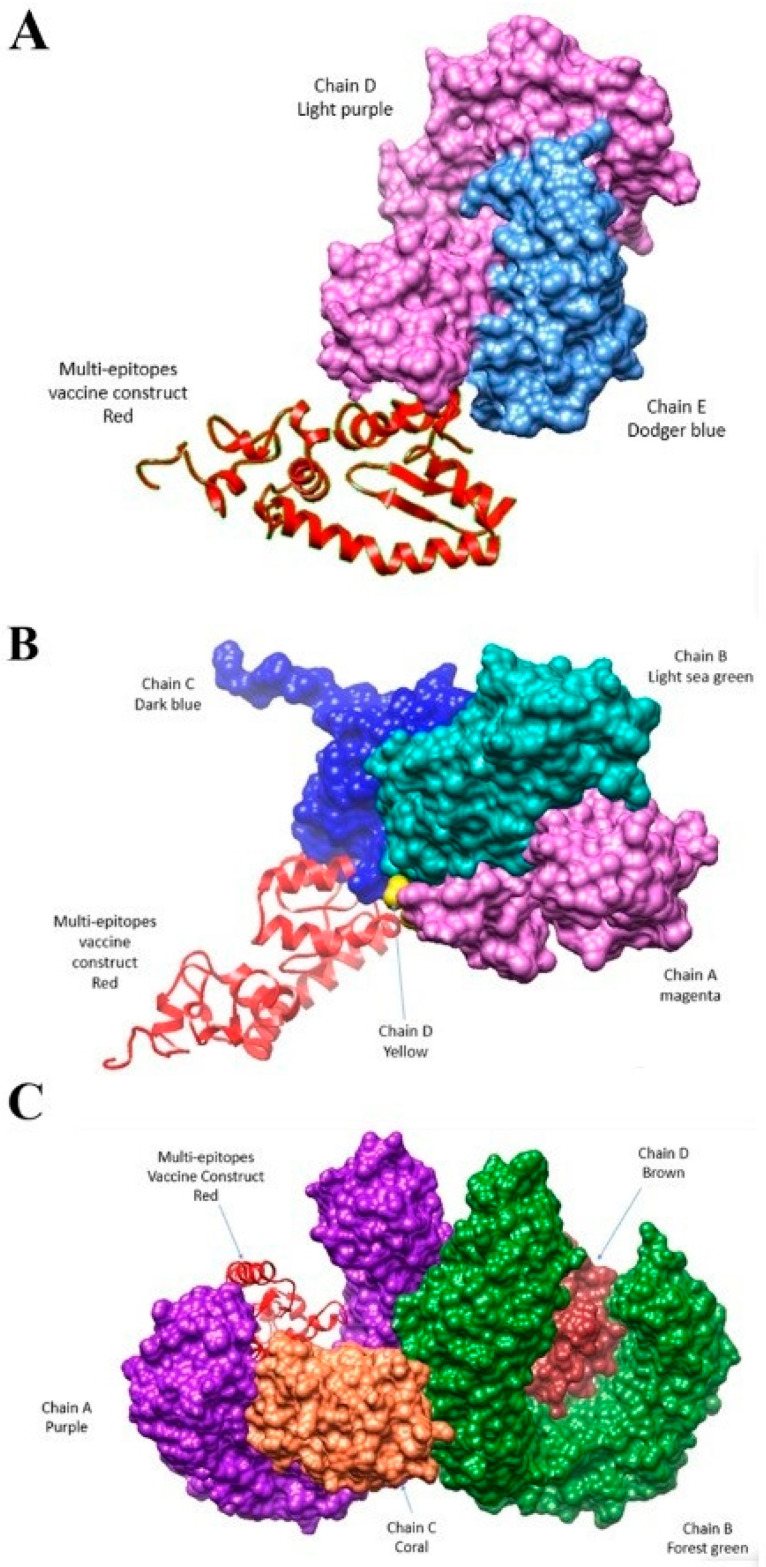
Docked complex of vaccine and MHC-I (**A**), MHC-II (**B**), and TLR-4 (**C**) immune cell receptors.

Molecular docking studies is a bioinformatics modeling approach which basically identifies interactions between ligand and receptors. Here, the designed vaccine molecule was checked for binding efficacy with MHC-I, MHC-II, and TLR-4 immune cell receptors. The results revealed that the designed vaccine construct has the ability to interact with immune cell receptors that can trigger proper immune responses against the targeted pathogen. Findings of the docking results, including cluster members and the lowest center energy score, are tabulated in Tables S2–S4, while the docked complexes are presented diagrammatically in Figure 9A–C.

**Figure 10:**
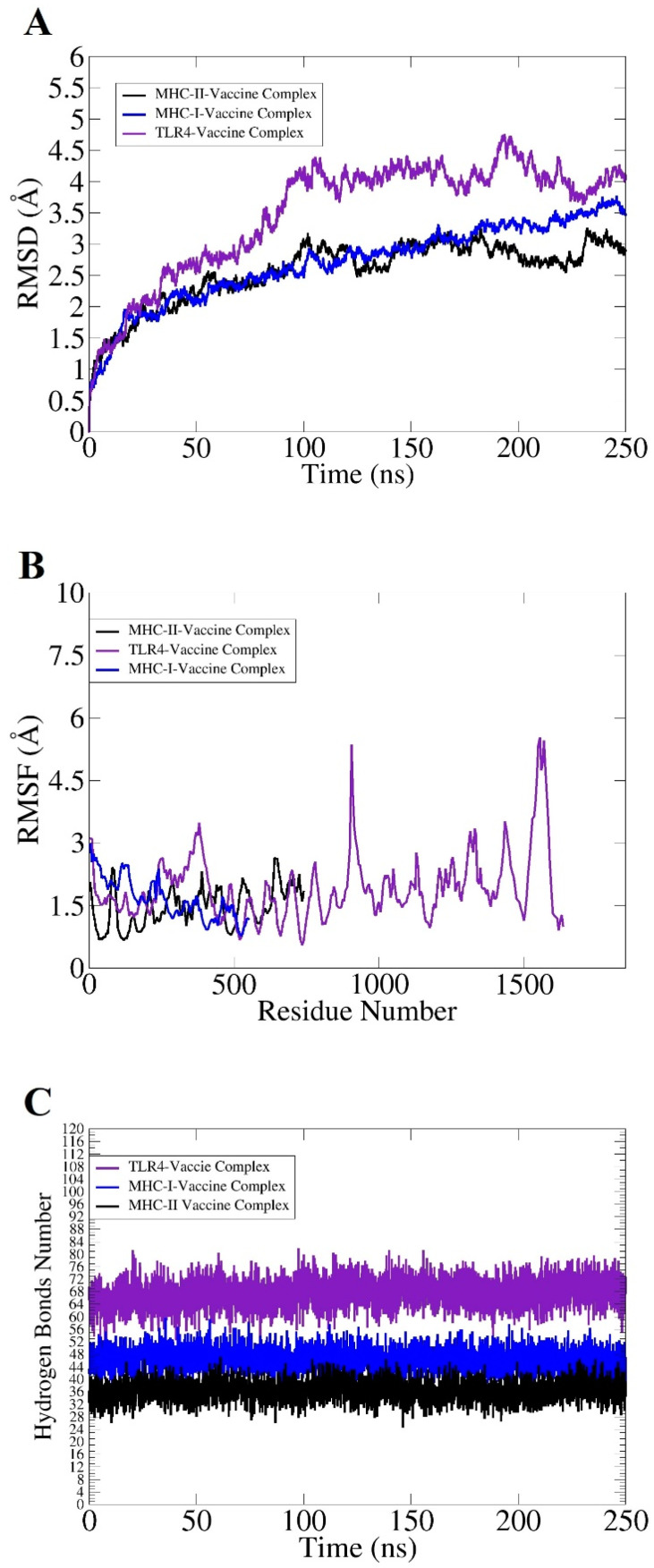
Statistical analysis of molecular dynamic simulation: (**A**) root mean square deviation (RMSD), (**B**) root square fluctuation (RMSF), and (**C**) hydrogen bonding analysis.

Among all docked complexes, the top complex of vaccine with MHC-I, MHC-II, and TLR-4 showed the lowest center energy of −594.1 kcal/mol, −706.7 kcal/mol, −787.2 kcal/mol, respectively, and were considered for the molecular dynamic simulation study. Molecular dynamics simulations evaluate the dynamic behavior of docked molecules. Molecular dynamics simulation analysis of all vaccine-immune receptor complexes show that no drastic changes occur throughout the simulation time, as indicated in Figure 10A–C. In root mean square deviation (RMSD) analysis, the TLR-4 construct showed good binding stability, followed by MHC-I and MHC-II. The graph shows small bit changes due to the presence of loops in the structure, but after 150 ns the system was found to be stable until the end of the simulations (Figure 10A). In the root mean square fluctuation (RMSF), vaccine-TLR-4 complex is 4.7 Å, vaccine-MHC-I is 3 Å, and vaccine-MHC-II complex showed an RMSF of 1.7 Å, as indicated in Figure 10B. Hydrogen bonding (H.B) is an attractive force between molecules that is very strong in nature compared with dipole-dipole forces [67]. Herein, several hydrogen bonds are formed between the designed vaccine and immune cell receptors, which indicates that there are strong interactions between vaccine and receptors. The TLR-4 and vaccine molecules produced more than 80 hydrogen bonds, while MHC-I and the vaccine formed hydrogen bonds between 56–60, and the vaccine and MHC-II molecules produced above 44 hydrogen bonds, as indicated in Figure 10C.

### 3.8. Free Binding Energy Estimation of Docked Molecules

In MMPB/GB-SA, interactions of vaccine and receptor molecules were investigated. Net free binding energy calculation is more reliable than the docking scoring approach. The MM-GBSA of vaccine-MHC-II is noted as −255.94 kcal/mol; for vaccine-MHC-I, it is −284.74 kcal/mol, and for TLR-4, it is −258.99 kcal/mol. On the other hand, the MM-PBSA net binding energy calculated for vaccine-MHC-II is −260.86 kcal/mol; for vaccine-MHC-I, it is −279.97 kcal/mol; and for vaccine-TLR-4, it is −260.93 kcal/mol. All the energy calculation values are tabulated in Table 4.

### 3.9. Interactive Residues of Vaccine-MHC-I, Vaccine MHC-II and Vaccine TLR-4

Interactions of vaccine are crucial to generate robust immune responses against a particular antigen. The designed vaccine construct showed interactions with several key residues of immune receptors. In Table 5, all the interactive residues of the vaccine to MHC-I, MHC-II and TLR-4 are indicated.

### 3.10. In Silico Host Immune Simulation

**Figure 11:**
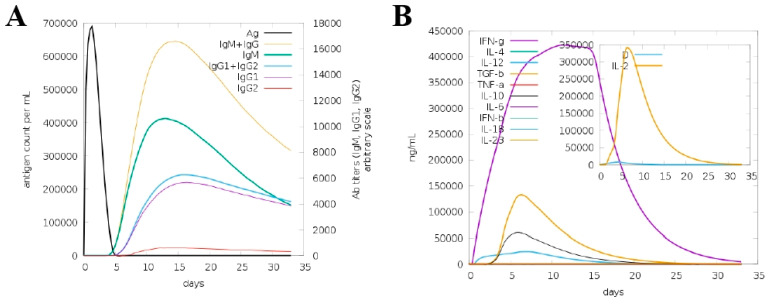
Different immune responses towards (MEV) against *E. hormaechei.* (**A**). Antibodies titer and (**B**) interferon and cytokines responses to the vaccine.

The overall finding of the host immune simulation showed that different immune responses are generated in the form of primary and secondary immune responses against the antigen. The immune responses against the pathogen consisted of different types, such as IgG and IgM, IgG1 + IgG2, IgG1 and IgG2. Also, an increase in interleukins production, as well different types of cytokines titers, was observed as indicated in Figure 11A,B. 

## 4. Conclusions

In conclusion, we present computational-based research work for the design of a multi-epitope-based vaccine construct against *E. hormaechei* by selecting conserved and antigenic proteins from the core proteome of the *E. hormaechei*. The selected proteins were used for B- and T-cell epitope prediction in order to generate cellular and humoral immune responses. The designed vaccine construct provokes both antibodies and cellular immune responses. The model vaccine also showed a maximum level of binding to MHC-I, MHC-II, and TLR-4 immune cell receptors, which can provide potent innate and adaptive immune responses for tackling the pathogen. Furthermore, the binding ability of the vaccine to immune cell receptors was confirmed by a molecular dynamics simulation approach. Several limitations exist that require further improvement and investigation in future research work i.e., the designed vaccine showed best immunogenicity, while the real immune responses against the targeted pathogen require validation by experimental studies. The criteria for selection and filtering the proteins targets for vaccine design were quite strict but still need to be further validated in vivo and in vitro. In conclusion, the designed vaccine model generates proper immune responses against *E. hormaechei* and can reduce the chances of infection, but experimentally evaluation is still required to uncover its real potency against *E. hormaechei*.

## Figures and Tables

**Table 1:** Selected proteins and predicted B-cells epitopes.

Protein Name/Accession Number	Predicted Epitopes
>core/8236/1/Org1_Gene1515 (curlin minor subunit (CsgB))	AAAGYDLANSEYNFAVNELSKSSFN
>core/5396/1/Org1_Gene1549 (flagellar basal-body rod protein (FlgF))	HPVVGEAGPIAVPEGAEITIA
	GSEVQRGDDDIFRLSAESQATRGPVLQADPT
>core/2987/11/Org11_Gene4825 (flagellar basal body P-ring protein (FlgI))	AGAQAGGSRVQVNQLNGG
	GTGDQTMQAPF
	NNVVSQPDTPLGGGQTVVVPQTDISVRDRGGSLQSVRSSTD

**Table 2:** Structural information for generated models of vaccine.

Model	Root Mean Square Deviation (RMSD)	MolProbity	Clash Score	Poor Rotamers	Rama Favored	GALAXY Energy
**Initial**	0.000	3.493	97.9	3.0	84.1	9003.96
MODEL 1	0.922	1.471	2.1	0.0	92.1	−3333.41
MODEL 2	0.880	1.517	2.5	0.8	92.1	−3327.95
MODEL 3	0.882	1.419	1.8	0.8	92.1	−3326.39
MODEL 4	0.786	1.494	2.5	0.0	92.7	−3318.02
MODEL 5	0.768	1.494	2.5	0.0	92.7	−3316.79
MODEL 6	0.804	1.517	2.5	0.0	92.1	−3315.93
MODEL 7	0.829	1.578	2.9	0.0	91.5	−3315.06
MODEL 8	0.874	1.572	3.2	0.0	92.7	−3312.58
MODEL 9	0.667	1.396	1.8	0.0	92.7	−3312.28
MODEL 10	0.831	1.517	2.5	0.8	92.1	−3310.59

**Table 3:** Pairs of mutated amino acids, Chi3 values and energy values.

Pairs of Amino Acid Residues	Chi3 Angle	Energy (kcal/mol)
LEU4-TYR-33	−74.84	3.02
LYS5-VAL-8	104.11	4.61
LEU13-LEU-29	118.91	3.55
ALA17-ASN-25	89.76	2.21
GLY21-ASN-25	91.77	4.63
PRO23-THR-40	−68.85	1.64
ILE26-ILE-38	115.25	6.87
LEU29-THR-36	−108.75	6.36
CYS30-THR-30	−67.54	2.88
ASN42-ILE-42	75.73	6.86
PHE46-ALA-59	123.39	7.39
GLU57-GLN-70	109.96	3.47
GLY75-HIS-78	122.15	4.19
LEU98-VAL-103	−80.03	1.94
ILE120-ALA-127	108.49	3.1
ALA127-MET-134	74.1	6.81
LYS133-TYR-136	105.58	2.38
GLY141-ALA-145	−85.33	6.8
ALA145-THR-148	114.28	2.59

**Table 4:** Computed net free binding energy of Vaccine-TLR-4, Vaccine-MHC-I and Vaccine-MHC-II complexes.

Energy Parameter	TLR-4-Vaccine Complex	Standard Deviation	MHC-I-Vaccine Complex	Standard Deviation	MHC-II-Vaccine Complex	Standard Deviation
**MM-GBSA**						
VDWAALS	−162.00	6.70	−184.87	7.36	−174.32	5.66
EEL	−71.36	2.67	−62.00	1.07	−49.52	2.08
Delta G gas	−233.36	7.25	−246.87	5.41	−223.84	6.43
Delta G solv	25.63	1.25	37.87	1.96	32.10	1.24
Delta Total	−258.99	8.36	−284.74	3.98	−255.94	7.93
**MM-PBSA**						
VDWAALS	−162.00	6.70	−184.87	7.36	−174.32	5.66
EEL	−71.36	2.67	−62.00	1.07	−49.52	2.08
Delta G gas	−233.36	7.25	−246.87	5.41	−223.84	6.43
Delta G solv	27.57	0.65	33.10	2.08	37.02	3.01
Delta Total	−260.93	7.64	−279.97	5.37	−260.86	9.31

**Table 5:** List of interactive residues MHC-I, MHC-II, and TLR-4 receptors.

Vaccine-Complexes	Interactive Residues
**Vaccine-MHC-I**	Ala31, Arg88, Asn65, Asn35, Ile45, Lys84, Glu32, Phe69, Met1, Tyr48, His34, Lys12, Lys3,Tyr29ser4 7, Pro140, Glu480
**Vaccine-MHC-II**	Asp28, Asp91, Arg97, Ala16, Phe46, Lys44, Tyr39, His115, Tyr97, Gly121, Lys83, Gln37, Thr22, Tyr18, Lys64, Lys3, Hhis34, Glu34, Thr27, Glu32
**Vaccine-TLR-4**	Asn526, Ala479, Asp379, Arg382, Lys477, Tyr451, Gln430, Ser381, Lys158, Lys420, Val338, Glu336, His334, Lys109, Lys477

## Data Availability

The data presented in this study are available within the article.

## Supplementary Materials

The following supporting information can be downloaded at: https://www.mdpi.com/article/10.3390/vaccines10050665/s1, Table S1: MHC class I and II predicted epitopes with their least predicted percentile score. Table S2: MHC class I and II predicted epitopes with their least predicted percentile score. Table S3: Docking results of Vaccine-MHC-II complex. Table S4: Docking results of TLR-4 and Vaccine
