# Supplementary material for: Designing of a Novel Multi-Antigenic Epitope-Based Vaccine against E. hormaechei: An Intergraded Reverse Vaccinology and Immunoinformatics Approach
Source: Vaccines (Basel). 2022 Apr 22;10(5):665. doi: 10.3390/vaccines10050665 (PMC9143018; doi:10.3390/vaccines10050665)
Supplement: Supplementary file 1 [file vaccines-10-00665-s001.zip › vaccines-1644014-supplementary.pdf]

Table S1. MHC class I and II predicted epitopes with their least predicted percentile score

MHC-I	P.S	MHC-II	P.S
YNFAVNELSK	2.1	YNFAVNELSKSS	0.37
AVNELSKSS	5.2		
AGYDLANSEY	0.14	AAAGYDLANSEY	14
AAAGYDLANS	22		
AGYDLANSEY	0.14	AAGYDLANSEYN	13
AAGYDLANSE	17		
ADMTPGKMDY	0.38	ADMTPGKMDYTS	44
TPGKMDYTS	3.9		
ADMTPGKMDY	0.38	GADMTPGKMDYT	52
GADMTPGKM	1.6		
GEAGPIAVP	0.51	VGEAGPIAVPE	1.71
EAGPIAVPE	4.8		
IAVPEGAEI	0.13	IAVPEGAEITIA	5.4
VPEGAEITIA	6.6		
VVGEAGPIAV	0.99	HPVVGEAGPIAV	2.5
HPVVGEAGPI	1.4		
RLSAESQAT	3.4	DIFRLSAESQAT	0.28
DIFRLSAES	3.1		
ATRGPVLQA	0.01	ATRGPVLQADPT	16
GPVLQADPT	2.1		
GPVLQADPT	5.7	GSEVQRGDDDIF	7
AQAGGSRVQV	0.33	GAQAGGSRVQVN	1.5
GAQAGGSRV	4.6		
GSRVQVNQL	0.42	GSRVQVNQLNGG	15
RVQVNQLNGG	16		
		AGAQAGGSRVQV	3.2

AQAGGSRVQV	0.33	VVVPQTDISVRD	18
AQAGGSRVQV	0.49		
RGGSLQSVR	0.79	RGGSLQSVRSST	27
SLQSVRSST	2.2		
VVSQPDTPL	0.86	NNVVSQPDTPLG	25
NNVVSQPDTP	23		
TPLGGGQTV	0.1	TPLGGGQTVVVP	3.2
GGGQTVVVP	43		
SLQSVRSST	2.2	GSLQSVRSSTD	s
GSLQSVRSS	3.7		

Table S2. Docking results of Vaccine-MHC-I complex

Cluster	Members	Representative	Weighted Score
**0**	151	Center	-594.1
		Lowest Energy	-688.3
**1**	68	Center	-656.9
		Lowest Energy	-692.2
**2**	63	Center	-646.3
		Lowest Energy	-708.3
**3**	61	Center	-665.9
		Lowest Energy	-782.6
**4**	50	Center	-679.9
		Lowest Energy	-730.0
**5**	44	Center	-687.9
		Lowest Energy	-687.9
**6**	43	Center	-594.8
		Lowest Energy	-648.0
**7**	35	Center	-589.3
		Lowest Energy	-722.6
**8**	31	Center	-723.8
		Lowest Energy	-723.8
**9**	31	Center	-657.0
		Lowest Energy	-711.9
**10**	27	Center	-587.2
		Lowest Energy	-659.9

Table S3. Docking results of Vaccine-MHC-II complex

Cluster	Members	Representative	Weighted Score
**0**	74	Center	-706.7
		Lowest Energy	-779.4
**1**	69	Center	-716.8
		Lowest Energy	-862.2
**2**	52	Center	-779.5
		Lowest Energy	-788.2
**3**	50	Center	-714.1
		Lowest Energy	-771.0
**4**	48	Center	-693.7
		Lowest Energy	-743.1
**5**	47	Center	-769.6
		Lowest Energy	-783.3
**6**	45	Center	-795.2
		Lowest Energy	-795.2
**7**	44	Center	-687.4
		Lowest Energy	-776.4
**8**	44	Center	-915.6
		Lowest Energy	-915.6
**9**	44	Center	-755.7
		Lowest Energy	-810.2
**10**	42	Center	-681.6
		Lowest Energy	-820.0

Table S4. Docking results of TLR-4 and Vaccine

Cluster	Members	Representative	Weighted Score
**0**	118	Center	-787.2
		Lowest Energy	-815.9
**1**	94	Center	-669.3
		Lowest Energy	-808.5
**2**	54	Center	-654.3
		Lowest Energy	-850.2
**3**	49	Center	-717.0
		Lowest Energy	-786.9
**4**	40	Center	-674.2
		Lowest Energy	-749.4
**5**	34	Center	-652.8
		Lowest Energy	-852.0
**6**	27	Center	-649.9
		Lowest Energy	-808.8
**7**	27	Center	-652.2
		Lowest Energy	-743.2
**8**	25	Center	-677.5
		Lowest Energy	-767.5
**9**	25	Center	-647.6
		Lowest Energy	-787.5
**10**	24	Center	-780.9
		Lowest Energy	-780.9
